# Treatment of hemopericardium caused by mitral balloon valvuloplasty with activated factor VII: a case report

**DOI:** 10.1186/1752-1947-8-33

**Published:** 2014-01-28

**Authors:** Jeremy Steele, Igor Mamkin, Berhane Worku, Iosif Gulkarov

**Affiliations:** 1 Department of Pediatrics, SUNY Upstate Medical University Center, Golisano Children’s Hospital, 750 East Adams Street, Syracuse, NY 13210, USA; 2 Division of Cardiology New York Methodist Hospital, 506 Sixth Street, Miner 3, Brooklyn, NY 11215, USA; 3 Department of Cardiothoracic Surgery, New York Methodist Hospital, 506 Sixth Street, Miner 3, Brooklyn, NY 11215, USA

**Keywords:** Complications and management, Factor VIIa, Hemopericardium, Mediastinal bleeding, Mitral balloon valvuloplasty

## Abstract

**Introduction:**

The use of mitral balloon valvuloplasty as a percutaneous intervention for mitral stenosis has been shown to be efficacious. Cardiac tamponade is a rare but serious complication of this procedure. Despite the low incidence of this event, cardiac tamponade is well-reported in the literature. The management strategy of this complication involves pericardial drainage and correction of coagulopathy, followed by surgical exploration if these interventions fail. With this case report, we demonstrate the successful application of activated factor VII in the management of bleeding after balloon valvuloplasty that persisted despite the standard treatments described above.

**Case presentation:**

Our patient was a 31-year-old Yemenite man with no significant past medical history, who presented with progressively worsening dyspnea on exertion and limited functional capacity over the last few years. A transesophageal echocardiogram revealed severe mitral stenosis, which was treated with a mitral valve valvuloplasty. The procedure was complicated by significant mediastinal bleeding that did not respond to routine maneuvers, which included pericardiocentesis and correction of coagulopathy. Our patient was evaluated for surgical intervention but responded to treatment with activated factor VII.

**Conclusion:**

Factor VII may be used in the treatment of refractory mediastinal bleeding secondary to mitral valvuloplasty prior to attempting surgical repair, and therefore may spare the patient the morbidity associated with surgery.

## Introduction

Cardiac perforation during cardiac catheterization procedures is very rare and only about one third result in some degree of cardiac tamponade [1-10]. The procedures responsible for the highest incidence of cardiac tamponade include percutaneous mitral valvuloplasty [11]. The incidence of cardiac tamponade during mitral balloon valvuloplasty (MBV) is reported to be between 1% and 9% [1-7]. The presence of cardiac tamponade is an absolute indication for emergent pericardial drainage and can be performed by either pericardiocentesis or surgical pericardiotomy [11,12]. Additional interventions that should be considered include correction of coagulopathy with protamine, fresh frozen plasma, cryoprecipitate and platelets, and resuscitation with blood transfusions and intravenous fluids [1,8,11,13,14]. These interventions are often successful in controlling bleeding within several hours [8]. Intractable bleeding requires a sternotomy. In this report, we describe the successful application of activated factor VII (aFVII) in the management of persistent bleeding during MBV, thereby avoiding a sternotomy and the associated morbidity of this approach.

## Case presentation

Our patient was a 31-year-old Yemenite man, with no significant past medical or surgical history, who was not on anticoagulants. He presented with progressively worsening dyspnea on exertion and limited functional capacity over the last few years. His physical examination was significant for an opening snap, a grade 2-3/6 diastolic murmur and a 1/6 holosystolic murmur. Laboratory parameters were significant for a normal coagulation profile. Our patient’s electrocardiogram showed left atrial enlargement and no evidence of ventricular hypertrophy. A transesophageal echocardiogram revealed normal left ventricular systolic function with an ejection fraction of 60% and left atrial dilation. The mitral valve was minimally calcified with mild thickening limited to the leaflet margin without chordal involvement. Characteristic echocardiographic features of rheumatic mitral stenosis were present with a Wilkins score of three [15]. The mean mitral gradient was 11mmHg and the valve area was 1.1cm^2^. Given our patient’s age, New York Heart Association Class III symptoms and mitral valve morphology, he was deemed to be a good candidate for MBV. Heparin was administered with an activated clotting time measured as 272 seconds. Valvuloplasty was performed utilizing a transeptal approach with a 26mm Inoue-balloon catheter.

At the end of the procedure, our patient was noted to have an effusion on the echocardiogram, with features of cardiac tamponade. He was otherwise hemodynamically stable. The effusion was treated with emergent pericardiocentesis and a pericardial catheter was left for continuous drainage. Pericardial catheter output ceased after the drainage of approximately one liter of blood. An hour after arrival to the intensive care unit, a pericardial effusion was noted again on surveillance transthoracic echocardiography. Our patient drained 350ml/h of bright red blood for the next three hours despite a normal coagulation profile. During this time, his presumed coagulopathy was corrected with protamine (25mg intravenously), two units of fresh frozen plasma, two single donor platelets and six units of cryoprecipitate, and his coagulation profile then rechecked. Complete evacuation of his pericardial sac was verified hourly with surveillance transthoracic echocardiography. Despite a normal coagulation profile the bleeding persisted, with its exact site of origin unknown. Our patient was warm, had normal acid-base status, and remained hemodynamically stable throughout, yet an additional 350cc of blood was drained within the next hour. At that time, the decision was made to administer 4.8mg (70μg/kg) of aFVII intravenously over two minutes. Drainage from the pericardial tube almost immediately ceased. Transesophageal echocardiography revealed resolution of the hemopericardium.

Our patient is doing well two years after the MBV. He has a mitral valve gradient of 4mmHg and he has functionally improved to New York Heart Association Class I. His coagulopathy work-up was negative.

## Discussion

To the best of our knowledge, this is the first report of successful use of aFVII in the management of intractable bleeding after MBV despite maximum medical therapy. An algorithm for management of postprocedural mediastinal bleeding usually involves placement of mediastinal drainage catheter and correction of coagulopathy. If bleeding is refractory and/or the patient becomes hemodynamically unstable, surgical intervention should be considered. In our case, our patient was hemodynamically stable yet had persistent bleeding that did not respond to the correction of coagulopathy and mediastinal drainage. Given our growing experience with use of aFVII in cardiac surgery and the relative stability of our patient, we decided to administer aFVII, with plans for transport to the operating room if bleeding did not subside. Drainage from the mediastinal tube almost immediately ceased after aFVII was administered.

Current indications for the use of aFVII are congenital factor VII deficiency, hemophilia and Glanzmann thrombasthenia. Despite its high cost (US$1 per microgram), there has recently been an influx of research citing increased off-label use of aFVII in the management of intractable bleeding, in not only cardiac surgery [16] but also intracranial hemorrhage, trauma, liver transplantation and prostatectomy [17]. Factor VII plays a very important role in the initiation of coagulation by combining with tissue factor (TF). Factor VII is constantly circulating in the bloodstream and is not exposed to TF until there is an injury to a blood vessel [16]. The activity of both factor VII and TF are required to initiate coagulation and this is accomplished when the TF/factor VIIa complex works in conjunction with the factor VIII/IX complex to activate factor X, which in turn is required for thrombin generation [16]. It has been shown that at certain dosing levels aFVII can bypass the requirement of factors VIII and IX and independently activate factor X. Additionally, it has been shown to normalize prothrombin times in patients with thrombocytopenia by improving platelet function [16]. The end effect is a thrombin burst and platelet activation that leads to a more effective platelet plug formation.

## Conclusion

aFVII may be considered prior to surgical intervention in hemodynamically stable patients in the context of mediastinal bleeding refractory to medical therapy after MBV.

## Consent

Written informed consent was obtained from the patient for publication of this case report. A copy of the written consent is available for review by the Editor-in-Chief of this journal.

## Abbreviations

aFVII: Activated factor VII; MBV: Mitral balloon valvuloplasty; TF: Tissue factor.

## Competing interests

The authors declare that they have no competing interests.

## Authors’ contributions

JS was involved in drafting the manuscript and revising it critically for important intellectual content. IM made substantial contributions to conception and design, and acquisition of data. BW was involved in revising the manuscript critically for important intellectual content. IG made substantial contributions to conception and design; was involved in drafting the manuscript and revising it critically for important intellectual content; and gave final approval of the version to be published. All authors read and approved the final manuscript.
